# A Pilot Survey of Clergy Regarding Mental Health Care for Children

**DOI:** 10.1155/2012/742410

**Published:** 2012-06-21

**Authors:** Leigh Blalock, Rachel E. Dew

**Affiliations:** ^1^Duke University Department of Psychiatry and Behavioral Sciences, Suite 100, 1901 N. Harrison Avenue, Cary, NC 27513, USA; ^2^Duke University Department of Psychiatry and Behavioral Sciences, Suite 300, 2608 Erwin Road, Durham, NC 27705, USA

## Abstract

Collaborations between healthcare and faith-based organizations have emerged in the drive to improve access to care. Little research has examined clergy views on collaborations in the provision of mental healthcare, particularly to children. The current paper reports survey responses of 25 clergy from diverse religious traditions concerning mental health care in children. Subjects queried include clergy referral habits, specific knowledge of childhood conditions such as depression and anxiety, past experiences with behavioral health workers, and resources available through their home institutions. Overall, surveyed clergy support collaborations to improve childhood mental health. However, they vary considerably in their confidence with recognizing mental illness in children and perceive significant barriers to collaborating with mental health providers.

## 1. Introduction 

As US healthcare costs continue to climb, large segments of the American population remain underserved. This is especially felt in the arena of behavioral health. In the struggle to improve access to care there has been a movement to form collaborations with faith-based organizations [1, 2]. However, much of this work has focused on medical illness, to the exclusion of behavioral health issues [3–6]. Mental health care, especially that for children, continues to be understaffed and patients underserved [7]. 

Collaborations between psychiatry and faith-based organizations pose unique problems [8]. The psychiatric establishment and religious organizations have historically viewed one another with suspicion and at times direct hostility [9, 10]. Religious organizations and clergy may be at odds with the medical establishment as to causes and proper treatment of such illnesses as depression and anxiety [11]. In some religious groups these maladies may be seen as stemming from a spiritual source rather than brain pathology [12]. 

Some research has emerged attempting to characterize the current relationship of psychiatry to the religious community. What little research is available on this topic largely relates to questions of adult mental health [11, 13, 14]. An analysis of the National Comorbidity Survey, a study of mental health in a large nationally-representative US sample, found that one-quarter of all respondents seeking mental health care sought this care from a clergy member [14]. The majority of those contacting clergy for mental health care in the last year saw no other providers. Nearly one-fourth of those contacting clergy for help met criteria for serious mental illness. Another study of urban and rural churches in the Southern US found that predominantly African-American churches provided more general health and mental health programs for their members than their Caucasian counterparts. Neither African-American or Caucasian churches had significant contact with or cross-referral with traditional healthcare agencies [10]. 

Despite evidence that a significant proportion of care for the mentally ill in America is provided by clergy, the literature on clergy views of mental health care and collaboration with mental health professionals is sparse. A 2007 paper by Leavey and colleagues reported on 32 interviews with male ministers, rabbi, and imams in the United Kingdom. A majority of respondents had received little or no training in mental health issues and felt low confidence in dealing with mentally ill members. Interviews indicated little specific knowledge of psychiatric disorders and a significant tendency to interpret symptoms such as psychosis as spiritual problems [2]. A 2008 survey of Protestant ministers in Hawaii found similar results, with the majority of respondents feeling undertrained for recognizing mental illness [4]. Interestingly, when presented with clinical vignettes, 40% of those endorsing inadequate mental health training said they would personally counsel the patients rather than referring to traditional psychiatric services. 

Attempts at rapprochement have been recently described. Training in spirituality and “cultural competence” have recently been added to education for psychiatric residents [15]. A 2012 report details an interdisciplinary training experience shared by psychiatric trainees and seminary students in South Carolina. Each group learned about the other's training and discussed barriers to and guidelines for collaboration. Pre- and postexperience surveys indicated an improvement in both groups opinion of the other's role in dealing with mental health problems [15]. 

Given that mental health services are severely lacking for children and adolescents, and that a large proportion of patients consult with clergy in preference to traditional mental health providers, collaborations with faith-based organizations to improve access to care should be explored. The following research study aimed to improve understanding of views of mental health care for children among clergy in the Southern United States. It also sought to assess this group's self-assessed understanding of childhood mental illnesses. Understanding the experience and attitudes of clergy toward mental health care can inform efforts to collaborate with faith-based organizations, which may ultimately improve provision of mental healthcare to US children.

## 2. Methods 

The authors collected names of religious institutions across religions within a 25-mile radius of Durham, N C. 2010 census data from the US Census Bureau indicate that residents of this area are 43% Caucasian, 41% African-American, and 5% Asian. 14% are identified as of Hispanic or Latino origin. 

Were the investigators able to obtain e-mail contact information for the institution, an e-mail was sent soliciting participation of the pastor or other institution leader in the anonymous online survey. This e-mail contained a link to the survey and a letter explaining the study aims and procedures. The survey was administered via Survey Monkey (http://www.surveymonkey.com/), a secure website. No identifying information was collected. The study was approved by the Duke University Medical Center Institutional Review Board.

A Likert-scale formatted questionnaire was created for the survey. Topics covered included questions related to perceptions of the need for mental health services among members; perceptions that clergy can influence their members with regard to seeking mental healthcare; to what degree clergy support referral of members to mental health services; clergy perceptions of their own knowledgebase regarding child mental health; opinions regarding collaboration; and barriers to collaboration with mental health professionals. 

Clergy reported demographic information (race, religious tradition) as well as information about the size of their organization. A series of questions examined what healthcare resources were available within the respondent's religious organization. Due to the small proportion of respondents (23%) to this pilot survey, as well as the relative demographic homogeneity of respondents, rigorous statistical comparison was deemed inappropriate and results were assessed using descriptive statistics only.

## 3. Results 

Results are displayed in Table 1. Of 108 survey invitations sent, 25 clergy responded and completed the online survey. Religious affiliations of the 108 institutions to which invitations were sent were as follows: Christian 91% (Protestant 83%, Catholic 7%), Jewish 6%, Muslim 2%, Hindu 1%, and other 1%. Religious affiliations of the 25 responders were as follows: Protestant Christians 86%, Jewish 7%, Hindu 4%, and Muslim 3%. Reported race of respondents was Caucasian 92%, African-American 4%, and South Asian or Indian 4%. Institutional size was quite varied, with 12% endorsing less than 100 members and 24% endorsing over 1000 members.

The majority of the sample (84%) agreed or strongly agreed that children in their institution were in need of mental health services. A majority also agreed or strongly agreed that the local community was in need of more mental health professionals caring for children. Most respondents (84%) felt that psychiatrists and psychologists had important roles in treating children, and nearly all stated that they would make a referral to a child psychiatrist if they thought it was needed.

Although the overall group endorsed openness to use of behavioral health services, 68% stated that they would prefer to personally counsel members rather than referring them to mental health professionals. 72% of polled clergy agreed that they held strong influence over the use of mental health services by their members; however, 60% disagreed that church members would ask them prior to seeking care from a mental health professional. A majority would support the use of psychiatric medication if recommended by a doctor, and most also supported the use of individual or family psychotherapy.

A series of questions queried the respondent's perception of his or her own knowledge about mental health in children. 60% agreed or strongly agreed that they felt knowledgeable about mental health disorders affecting children while 48% felt confident in their ability to recognize the need for mental health services for children. 76% endorsed knowledge of where to refer a child or family for mental health care. 

The clergypersons were then asked about their perception of knowledge regarding specific psychiatric disorders in children. A slight majority felt they did not know enough about depression in children. A similar breakdown was seen for knowledge regarding anxiety disorders in children, attention deficit hyperactivity disorder, and childhood substance abuse. As a group the sample felt less confident in their knowledge about childhood bipolar disorder, autism, schizophrenia, oppositional defiant disorder, and conduct disorder. 70% of those surveyed agreed or strongly agreed that they would like to know more about the specific psychiatric disorders mentioned in the survey. 

The survey went on to query opinions about collaborations between faith-based organizations and mental health professionals. All respondents agreed or strongly agreed that it is valuable to collaborate with mental health professionals; however, 48% felt that their role as a spiritual leader in caring for the mentally-ill was not valued by mental health professionals. 84% agreed or strongly agreed that they would prefer to send members to a counselor sensitive to spiritual needs, and the majority endorsed an urge to educate mental health professionals on the unique spiritual beliefs, values and needs of their members. When asked about previous experiences in working with mental health professionals 84% endorsed having had good experiences in such collaborations. However, 61% endorsed bad experiences in working with behavioral health workers.

The survey asked respondents to reflect on barriers to finding adequate psychiatric services for children in their institutions. All specifically listed barriers were perceived by a majority of respondents. These included problems with uninsured children, lack of mental health professionals available, lack of clarity about where to refer, lack of confidence in determining whether mental health services were needed, and lack of understanding of spiritual needs among mental health professionals. About half the sample endorsed feeling that their members do not want to see mental health professionals.

Healthcare resources available at the respondents' home institutions were variable. 52% felt that programming for youth at their institution was inadequate, whereas 56% felt their parental support groups were insufficient. Most did not feel there were adequate on-site services for counseling, and the majority did not have a parish nurse or health coordinator in house. Most would like to collaborate to increase support for youth and parents among their parishioners.

The final section of the survey allow for additional comments. Comments offered included the following.

* “I have trouble locating mental health professionals for referrals that support values and attitudes that are consistent with Christian faith. I would like to have a way to connect with mental health professionals that are Christians by specialty.”*

*“I'm tired of our church members being disrespected for their beliefs and the choices they make that are shaped by their faith.” *

*“Clergy need to distinguish between pastoral care and pastoral counseling. Some are not trained for both and ought not to attempt counseling beyond having some basic referral skills.”*

*“Mental health professionals need to stop seeing spirituality is politically incorrect for counseling. Patients with active church involvement need to see how to integrate their faith into their mental health.”*

*“Sometimes the clergy and mental health professional ought to collaborate (with patient's permission) but this is rarely encouraged.”*

## 4. Discussion 

Based on these results, the current group of clergy showed significant acceptance of behavioral health care for children from their institutions. Yet, the group also seemed to endorse cautious feelings in considering this topic. They admitted to seeing many barriers to collaboration, and having faced stigma from the psychiatric community. Most expressed a preference for personally providing counseling to members of their religious institutions, rather than referring them to mental health providers. Comments revealed an urge to identify clinicians that would be respectful of members belief systems and implied that members of their institutions had faced difficulty in integrating their faith with standard mental health treatment. Furthermore, a large part of the sample felt their membership would not want to work with psychiatrists or other mental health providers. 

Our results conform with previous studies, [10, 11, 13, 15], in that those surveyed felt generally positive toward collaborating with the mental health establishment, but also endorsed hesitance related to stigma felt by themselves and their members. As in the Hawaiian study [13], although a significant proportion admitted to inadequate knowledge of psychiatric disorders in children, a majority would rather counsel members personally than refer them out.

In contrast to previous clergy surveys [11, 13], nearly half of this sample felt confident in their knowledge of common psychiatric disorders. The majority, however, endorsed a desire to learn more about less familiar mental illnesses. Educational efforts are likely to be hampered by the presence of stigma and disrespect clergy sometimes feel when dealing with mental health workers. However, it may be that mutual education between the two groups can be successful [15]. 

## 5. Limitations

The presented study has several features that limit its generalizability. First, the sample was not randomly selected. Second, response rate was low. Thirdly, the survey was created by the investigators without a previous validation study. Fourth, respondents were all based in the Southern US and were predominantly Caucasian and Protestant. Fifth, this study explores the subject of psychiatry and religion from the point of view of clergy with no input from mental health workers.

Much further work in this area is needed to fully understand how collaboration between religious leaders and the mental health establishment could be accomplished and what barriers need to be addressed. Further research should involve more qualitative methodology to create surveys followed by validation studies. Investigators may then seek to confirm findings in a larger, more representative sample. Future research should explore viewpoints from multiple stakeholder groups to facilitate active dialogue between traditional mental health providers and religious organizations. 

## Figures and Tables

**Table 1:** Survey items and responses (*N* = 25).

Item	Strongly	Agree	Disagree	Strongly
	agree (%)	(%)	(%)	disagree (%)
*Perceptions of need of mental health services in their congregation *				
There is need for mental health (MH) services for children in my congregation.	16	68	8	8
My community needs more MH professionals to care for children.	36	40	16	8
Psychiatrists and psychologists have important roles in treating children.	40	44	12	4
I would prefer to counsel my members rather than refer them to MH professionals.	8	16	40	36
I would refer a child to a psychiatrist if I felt they needed it.	52	44	0	4

*Perception of influence*				
I have a lot of influence on whether my members seek MH services.	0	72	24	4
I support treatment with psychiatric medication if recommended by an MD.	20	76	4	0
I support a member seeking “talk therapy” from an MH professional.	29	58	13	0
I support a member seeking family therapy or counseling.	40	56	4	0
My members ask me before they seek MH treatment elsewhere.	4	28	68	0

*Perception of knowledge base*				
I feel knowledgeable about MH disorders affecting children.	8	52	36	4
I feel confident in my ability to know if a child needs MH services.	4	44	52	0
I know where to refer a child and family if they need MH services	12	64	20	4
I know enough about…ADHD	17	42	37	4
Depression in children	4	44	48	4
Anxiety in children	4	44	52	0
Bipolar disorder in children	4	28	64	4
Autism	4	28	64	4
Schizophrenia in children	4	28	64	4
Substance abuse in children	12	44	40	4
Disruptive behavior disorders in children	8	40	48	4
Oppositional defiant disorder	8	24	64	4
Conduct disorder	8	36	52	4
I would like to know more about the above disorders.	13	57	26	4

*Perception of collaboration with MH professionals*				
It is valuable to collaborate with MH professionals.	32	68	0	0
MH professionals value my role as a spiritual leader in caring for the mentally ill.	16	36	44	4
I prefer to send my members to a counselor who is sensitive to spiritual needs.	44	40	16	0
I would like to educate MH professionals on the unique spiritual beliefs, values, and needs of my members.	32	56	12	0
I have had a good experience with MH professionals.	12	72	16	0
I have had a bad experience with MH professionals.	4	57	35	4

*Perception of barriers*				
There are many barriers for children who need adequate mental health services in my community.	28	52	20	0
*The following are large barriers*				
no insurance,	36	48	16	0
not enough MH professionals in my community,	12	48	36	4
unclear where to refer children in need,	16	52	32	0
unclear how to determine if a child needs MH services.	24	40	36	0
MH professionals do not understand the spiritual needs of my members.	16	48	36	0
My members do not want to see MH professionals.	8	44	44	4

*Resources at religious institutions*				
We have enough youth programs at my institution.	8	40	40	12
We have plenty of parental support groups.	4	40	48	8
We have adequate services for counseling on site.	0	28	60	12
We have a parish nurse or health coordinator on site.	0	16	68	16
I want to collaborate with others to increase the youth groups program.	4	60	32	4
I want to collaborate with others to increase the parental support program.	4	60	28	8
